# Clusterin/Apolipoprotein J Attenuates Angiotensin II-Induced Renal Fibrosis

**DOI:** 10.1371/journal.pone.0105635

**Published:** 2014-08-22

**Authors:** Gwon-Soo Jung, Jae-Han Jeon, Yun-A Jung, Yeon-Kyung Choi, Hye-Soon Kim, Jung-Guk Kim, Keun-Gyu Park, Mi-Kyung Kim, In-Kyu Lee

**Affiliations:** 1 Division of Endocrinology and Metabolism, Department of Internal Medicine, Kyungpook National University School of Medicine, Daegu, Republic of Korea; 2 Division of Endocrinology and Metabolism, Department of Internal Medicine, Keimyung University School of Medicine, Daegu, Republic of Korea; The University of Manchester, United Kingdom

## Abstract

The blockade of angiotensin II (Ang II) is a major therapeutic strategy for diabetic nephropathy. The main roles of Ang II in renal disease are mediated via the Ang type 1 receptor (AT1R). Upregulation of clusterin/apolipoprotein J has been reported in nephropathy models, suggesting it has a protective role in nephropathogenesis. Here, we studied how clusterin acts against Ang II-induced renal fibrosis. Levels of AT1R and fibrotic markers in clusterin-/- mice and Ang II infused rats transfected with an adenovirus encoding clusterin were evaluated by immunoblot analysis, real time RT-PCR, and immunohistochemical staining. The effect of clusterin on renal fibrosis was evaluated in NRK-52E cells, a cultured renal tubular epithelial cell line, using immunoblot analysis and real time RT-PCR. Nuclear localization of NF-κB was evaluated using immunofluorecence and co-immunoprecipitation. Renal fibrosis and expression of AT1R was higher in the kidneys of clusterin^-/-^ mice than in those of wild-type mice. Furthermore, loss of clusterin accelerated Ang II-stimulated renal fibrosis and AT1R expression. Overexpression of clusterin in proximal tubular epithelial cells decreased the levels of Ang II-stimulated fibrotic markers and AT1R. Moreover, intrarenal delivery of clusterin attenuated Ang II-mediated expression of fibrotic markers and AT1R in rats. Fluorescence microscopy and co-immunoprecipitation in conjunction with western blot revealed that clusterin inhibited Ang II-stimulated nuclear localization of p-NF-κB via a direct physical interaction and subsequently decreased the AT1R level in proximal tubular epithelial cells. These data suggest that clusterin attenuates Ang II-induced renal fibrosis by inhibition of NF-κB activation and subsequent downregulation of AT1R. This study raises the possibility that clusterin could be used as a therapeutic target for Ang II-induced renal diseases.

## Introduction

Renal fibrosis, mainly characterized by extracellular matrix (ECM) proteins deposition, is the universal mechanism of chronic kidney disease [1], [2]. Angiotensin II (Ang II) contributes to the development of renal fibrosis by upregulating profibrotic factors and inducing epithelial-mesenchymal transition [3]. It has been shown that in cultured renal cells, Ang II induces protein expressions which mainly play roles in cellular growth and matrix formation [4]; this effect is mainly mediated by the release of transforming growth factor β (TGF-β) [5] and this process can be partially attenuated by Ang-converting enzyme (ACE) inhibitors and Ang type 1 (AT1) antagonists [6], [7]. Furthermore, Ang II is involved in recruitment of inflammatory cells and increases the expression levels of chemokines, adhesion molecules, cytokines, and other growth factors [8], [9]. ACE inhibitors and AT1 antagonists ameliorate kidney disease progression in humans and animal models by reducing proteinuria, inflammatory cell infiltration and fibrosis [10], [11]. Ang II is involved in the activation of a number of transcription factors as well, such as NF-κB, members of the signal transducer and activator of transcription family and activator protein-1. NF-κB is an ubiquitous transcription factor involved in immune reactions, inflammation, proliferation, apoptosis and tumorigenesis [12]. As its role in a profinflammatory signal is well established, the involvement of NF-κB in pathologic renal conditions such as nephritis, tubulointerstitial disorders and proteinuria has also been widely investigated [13], [14]. Moreover, recently, it has been found that NF-κB is a key upstream mediator of diabetic nephropathy which is provoked by multiple pathophysiologies such as inappropriate hyperactivation of Ang II, increased synthesis of advanced glycation end products and reactive oxygen species [13], [15], [16].

Clusterin/apolipoprotein J is a glycoprotein expressed ubiquitiously in most human tissues and presents as two isoforms: one is a predominant conventional heterodimeric secretory form whereas the other is a nuclear form [17], [18]. Clusterin is implicated in a variety of physiological processes, including apoptosis, inflammation, lipid transportation, cell-to-cell interactions and aging; and additionally, it plays roles in pathological disorders demonstrated by increased levels in neurodegenerative disorders, ischemic heart disease, malignancies and diabetic conditions [19], [20]. Several previous reports have proven a beneficial role of clusterin in preventing progressive glomerulopathy and mesangial cell injury [21], [22]. A recent study also showed that clusterin attenuates renal fibrosis in a mouse model of unilateral urethral obstruction (UUO) [23]. These results suggest that clusterin protects kidney from fibrosis. Therefore, herein, we focused on the role of clusterin in Ang II-induced renal fibrosis, which is more relevant to the pathophysiology of renal diseases.

## Materials and Methods

### Reagents and plasmids

The recombinant human Ang II was purchased from Sigma (St. Louis, MO). The anti-plasminogen activator inhibitor-1 (PAI-1) and anti-fibronectin antibodies were purchased from BD Biosciences (San Jose, CA). The anti-collagen type I and anti-GFP antibodies were purchased from Abcam (Cambridge, UK). The anti-actin antibody was purchased from Sigma. The anti-clusterin and anti-phospho-Smad3 antibodies for immunohistochemical staining were purchased from Santa Cruz Biotechnology (Santa Cruz, CA). Anti–PAI-1 and anti-fibronectin antibodies were purchased from BD Biosciences (San Jose, CA). Anti–collagen type I antibody was purchased from Abcam (Cambridge, UK). Anti-phospho-NF-κB p65, anti-phospho-NF-IκBα, anti-IκBα and anti-phospho-Smad3 antibodies for immunoblot analysis were purchased from Cell Signaling Technology (Beverly, MA). The cDNA encoding rat clusterin was purchased from Benebiosis (Seoul, Korea).

### Animals

Male 8-week-old Sprague-Dawley rats and male 8-week-old C57BL/6 mice were purchased from Samtako (Osan, Korea) as described previously [23]. Clusterin knockout (Clu^-/-^) mice on a C57BL/6 genetic background were generated as described previously [23].

### Cell culture

The NRK-52E rat renal proximal tubular epithelial cell line was purchased from the American Type Culture Collection (Manassas, VA) and cultured as described previously [23]. The cells were then rendered quiescent by incubation for 24 h in medium containing 0.5% FBS, infected with Ad-clusterin in serum-free medium for 2 h, and then cultured in medium containing 0.5% FBS. After incubation for a further 20 h in medium containing 0.5% FBS, the cells were incubated with 200 nM Ang II for 8 h. The cells were processed as described below.

### Experimental infusion of Ang II and in vivo infection

Alzet osmotic mini-pumps (ALZA Scientific Products, Mountain View, CA) were implanted subcutaneously into Sprague-Dawley rats and Clu^-/-^ C57BL/6 mice. The pumps delivered saline (vehicle) or Ang II at a rate of 200 ng/min/kg (rats) or 1 µg/min/kg (mice) for 14 days. Viral infection was performed as previously described [23]. 14 days after Ang II infusion and adenovirus infection, the rats and mice were euthanized with an intraperitoneal injection of pentobarbital (50 mg/kg; Entobar, Hanlim Pharm. Co., Yongin, Korea) and their kidneys were removed and embedded in paraffin for histologic examinations as described earlier [23].

### Generation of recombinant adenovirus

Recombinant adenovirus was generated as described previously [23].

### Quantitative real-time RT-PCR

Total RNAs were obtained from NRK-52E cells and rat or mouse kidneys using Trizol Reagent (Invitrogen, Carlsbad, CA), according to the manufacturer's instructions. The cDNAs were synthesized using a first-strand cDNA kit (Fermentas, Hanover, MD). Quantitative real-time RT-PCR was performed using the SYBR Green PCR Master Mix Kit (Applied Biosystems, Warrington, UK) and the StepOnePlusTM Real-Time PCR System (Applied Biosystems). The thermal cycling conditions were as follows: 95°C for 10 min, followed by 40 cycles of 95°C for 15 s and 60°C for 1 min. The sequences of the primers, which were designed using AB StepOne software (v2.1) and were based on the relevant sequences deposited in GenBank, were as follows: rat PAI-1 (GenBank accession NM_012620.1: sense, 5′-CACCCCTTCCAGAGTCCCATA-3′; antisense, 5′-GCTGAAACACTTTTACTCCGAAGTT-3′), rat type 1 collagen (GenBank accession NM_053304.1: sense, 5′-GTGCGATGGCGTGCTATG-3′; antisense, 5′-TCGCCCTCCCGTTTTTG-3′), rat fibronectin (GenBank accession NM_019143.2: sense, 5′-ACCTGCAAGCCAATAGCTGAGA-3′; antisense, 5′-CCAGCCTTGGTAGGGCTTTT-3′), rat AT1R (GenBank accession NM_030985.4: sense, 5′-CAAGTCCCACTCAAGCCTGTCT-3′; antisense, 5′-TGTTATCCGAAGGCCGGTAA-3′),), rat clusterin (GenBank accessions NM_053021.2: sense, 5′- GGGAAGAGTGTAAGCCCTGC-3′; antisense; 5′-CGAGTGAAGCTGTCCTGCAT-3′), rat GAPDH (GenBank accession NM_017008.4: sense, 5′-TGCCGCCTGGAGAAACC-3′; antisense, 5′-AGCCCAAGGATGCCCTTTAGT-3′), mouse PAI-1 (GenBank accession NM_008871.2: sense, 5′-AATCCCACACAGCCCATCA-3′; antisense, 5′-GGACCACCTGCTGAAACACTTT-3′), mouse type 1 collagen (GenBank accession NM_007742.3: sense, 5′-GCCTTGGAGGAAACTTTGCTT-3′; antisense, 5′-GCACGGAAACTCCAGCTGAT-3′), mouse fibronectin (GenBank accession NM_010233.2: sense, 5′-GATATCACCGCCAACTCATTCA-3′; antisense, 5′-CAGAATGCTCGGCGTGATG-3′), mouse GAPDH (GenBank accession NM_008084.2: sense, 5′-GAAGGGTGGAGCCAAAAG-3′; antisense, 5′-GCTGACAATCTTGAGTGAGT-3′ mouse clusterin (NM_013492.2: sense, 5′-TGGACACAGTGGCGGAGAA-3′; antisense, 5′-CATTCCGCAGGCTTTTC-3′). Reaction specificity was confirmed by melting curve analysis. The housekeeping gene GAPDH was used as an internal standard.

### Immunoblot analysis

The cells were washed twice with phosphate-buffered saline (PBS) and suspended in RIPA buffer. The cells were then lysed on ice for 30 min and the cell lysate was collected by centrifugation at 15000×*g* for 10 min. Proteins were quantified using a protein assay kit (Bio-Rad, Richmond, CA). Thirty micrograms of cell lysate were separated by SDS-PAGE and electro-transferred onto a PVDF membrane (Millipore Corporation, Bedford, MA). The membrane was blocked with 5% skimmed milk in TBS containing 0.1% Tween 20 for 1 h and then incubated with the anti-clusterin (1∶3000), anti-PAI-1 (1∶3000), anti-type 1 collagen (1∶1000), anti-fibronectin (1∶1000), anti-phospho-NF-κB (1∶1000), or anti-AT1R (1∶1000) or anti-phopho-Smad3 (1∶1000) polyclonal antibody at 4°C with gentle shaking overnight. The membrane was then washed three times in TBS containing 0.1% Tween 20 for 10 min. The antibodies were detected using a horseradish peroxidase-linked secondary antibody (Santa Cruz) and the ECL Western Blotting Detection System (Amersham, Buckinghamshire, UK). The membrane was re-blotted with an anti-actin antibody to verify equal loading of the protein in each lane.

Nuclear extracts were isolated from cells using the NucBusterTM Protein Extraction Kit (Calbiochem, LA Jolla, CA), according to the manufacturer's instructions. For NF-κB analyses, the cytoplasmic and nuclear extracts were incubated with an anti-phospho-NF-κB (1∶1000) antibody (Cell Signaling Technology). Densitometric measurements of the bands were performed using the digitalized scientific program UN-SCAN-IT (Silk Scientific Corporation, Orem, UT).

### Co-immunoprecipitation assay

The cells were washed once in PBS and lysed on ice in RIPA buffer. The cell extract was centrifuged at 4°C for 10 min at 13,000 rpm. An aliquot of the cleared extract was retained as the input fraction and the remainder was used for co-immunoprecipitation. Five-hundred micrograms of nuclear extracts prepared from NRK-52E cells were mixed with 20 µl of protein A/G PLUS agarose (Santa Cruz) in RIPA buffer and incubated at 4°C for 1 h with gentle agitation. The mixture was then centrifuged for 1 min at 3000 rpm for pre-clearing. The recovered supernatant was incubated with anti-phospho-NF-kB antibody (1∶50) at 4°C overnight with mild shaking and then 40 µl of protein A/G PLUS agarose was added and the incubation was continued for a further 3 h at 4°C with gentle shaking. The protein A/G-precipitated protein complex was recovered by centrifugation and then washed three times with immunoprecipitation assay buffer. The samples were then analyzed by immunoblotting with an anti-clusterin antibody.

### Immunofluorescence

The cells were washed with ice-cold PBS, fixed with 4% paraformaldehyde for 10 min at room temperature, and then permeabilized with 0.1 M glycine and 0.1% Triton X-100. The cells were immunostained with an anti-phospho-NF-κB (1∶100) and anti-clusterin (1∶100) antibody overnight at 4°C. The cells were then washed three times with PBS (5 min per wash) and incubated with Cy3-conjugated secondary antibody (Jackson Immunoresearch, West Grove, PA) and Alexa Fluor 488-conjugated secondary antibody (Invitrogen, Karlsruhe, Germany) at room temperature for 3 h. DNA was stained with Hoechst 33342 (Pierce Chemical Company, Rockford, IL). The cells were examined by fluorescence microscopy (Olympus America Inc., Center Valley, PA).

### Histological analysis

Histological analyses were performed as described previously [23]. Immunohistochemical staining was performed by incubating the kidney sections with anti-GFP (1∶250), anti-clusterin (1∶100), anti-PAI-1 (1∶250), anti-type I collagen (1∶250), anti-fibronectin (1∶250), anti-AT1R (1∶250) and p-Smad3 (1∶250) primary antibodies, followed by horseradish peroxidase-conjugated anti-mouse or anti-rabbit IgG secondary antibodies (Dako, Glostrup, Denmark).

### Ethics statement

All procedures relevant with experiments were performed according to the appropriate institutional guidelines for animal research. The protocol gained approval of the Committee on the Ethics of Animal Experiments of the Keimyung University School of Medicine (Permit Number: KM-2011-10)

### Statistical analyses

All data were analyzed by analysis of variance followed by a post hoc least significant difference test. The data were expressed as the mean ± SEM. *P*<0.05 was considered statistically significant. At least three independent tests per experiments were performed.

## Results

### Loss of clusterin increases Ang II-induced renal fibrosis and expression of PAI-1, type I collagen, fibronectin and p-Smad3

**Figure 1:**
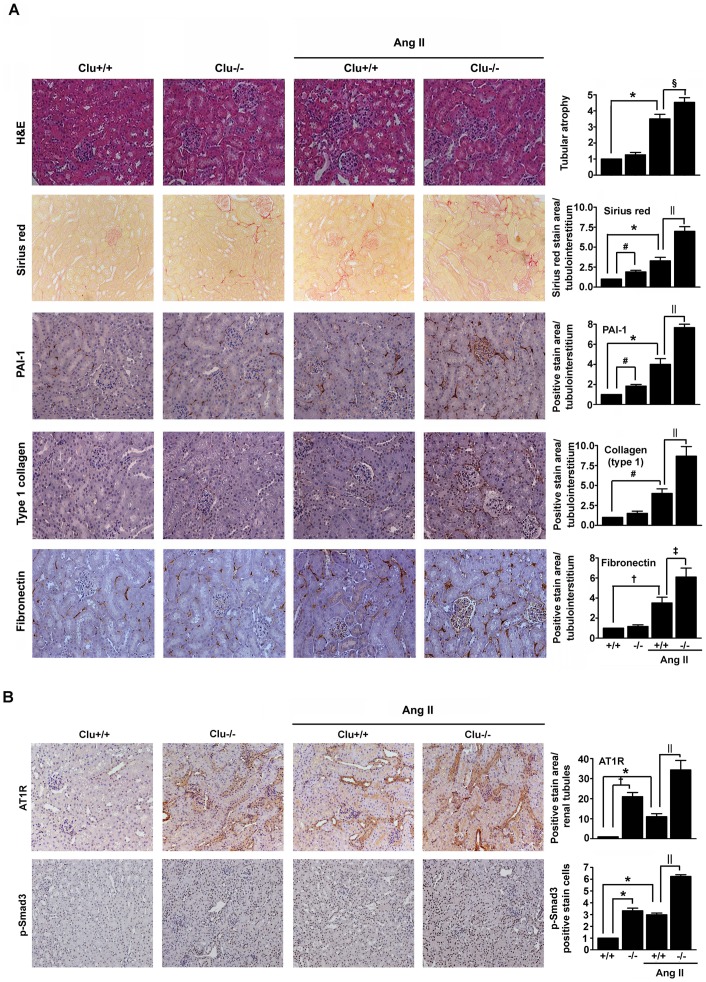
Effects of knockout of clusterin on Ang II-induced renal fibrosis. Representative images of renal cortex sections from wild-type (Clu^+/+^) and Clu^-/-^ mice treated with or without Ang II for 14 d. (**A**) The sections were stained with H&E or Sirius red, or were immunostained with antibodies targeting PAI-1, fibronectin, and type I collagen. The number of atrophic tubules was determined by measurement of the abnormal irregular and dilated tubular basement membranes in five random fields of H&E-stained sections under high-power magnification. Areas of positive staining with Sirius red and positive immunostaining with PAI-1, fibronectin, and type I collagen antibodies were quantified by computer-based morphometric analysis. (**B**) The sections were immunostained with an antibody targeting AT1R and p-Smad3. Areas of positive immunostaining were quantified by computer-based morphometric analysis. Data were normalized to the untreated wild-type and are expressed as the mean ± SEM of n = 5 random fields of each kidney (n = 5 in each group). ^*^ *P*<0.01, ^#^ *P*<0.05, and ^†^ *P*<0.001 compared with untreated wild-type mice and ^‡^ *P*<0.01, ^§^ *P*<0.05, and ^||^ *P*<0.001 compared with Ang II-treated wild-type mice. Original magnification, ×200.

Osmotic mini pumps that delivered Ang II at a rate of 1 µg/min/kg were implanted into the subcutaneous space of wild-type and Clu^-/^) C57BL/6 mice through an incision in the posterior neck. After exposure to Ang II for 14 d, the mice were euthanized and renal cortex sections were analyzed. The Ang II treatment increased tubular atrophy and renal fibrosis in both groups of mice, and Ang II-treated Clu^-/-^ mice exhibited significantly higher levels of renal tubulointerstitial damage and fibrosis than Ang II-treated wild-type mice (Fig. 1A). Furthermore, immunohistochemical staining revealed that the expression levels of PAI-1, type I collagen, and fibronectin were significantly higher in the kidneys of Ang II-treated Clu^-/-^ mice than those of Ang II-treated wild-type mice (Fig. 1A). Renal expression of the AT1 receptor (AT1R) was significantly higher in Clu^-/-^ mice than wild-type mice and loss of clusterin enhanced Ang II-stimulated AT1R expression (Fig. 1B). Expression of phosphorylated Smad3 (p-Smad3) was significantly higher in the kidneys of Clu^-/-^ mice, and was higher than that in wild-type mice after Ang II stimulation (Fig. 1B). These data indicate that clusterin is involved in Ang II-induced renal fibrosis.

### Adenovirus-mediated overexpression of clusterin inhibits Ang II-stimulated expression of PAI-1, type I collagen, fibronectin and p-Smad3

**Figure 2:**
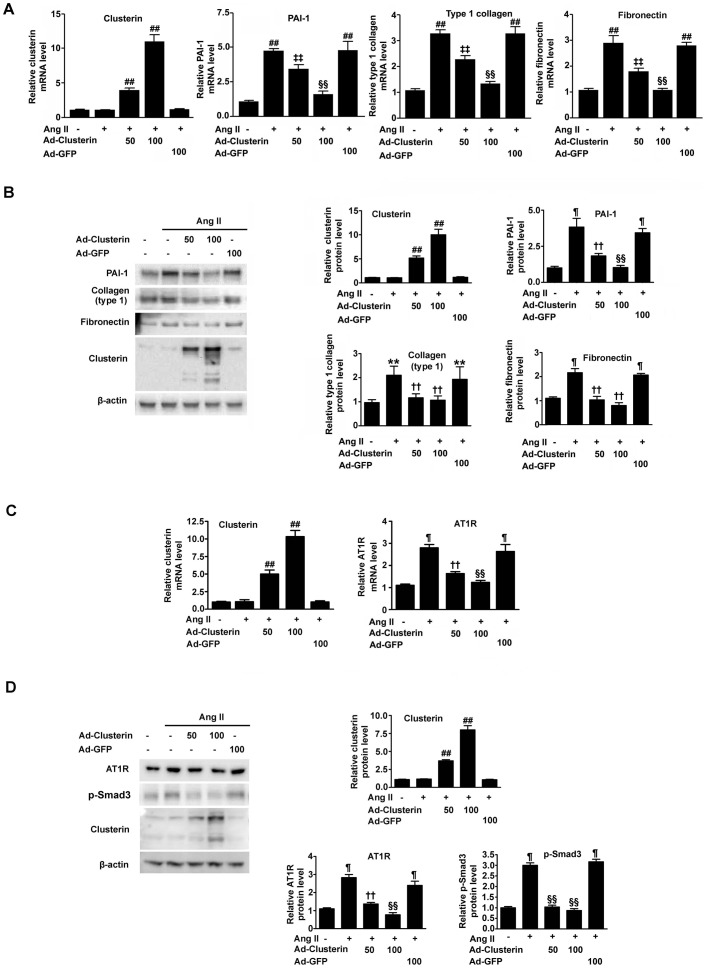
Effects of clusterin on Ang II–stimulated expression of PAI-1, type 1 collagen, and fibronectin. Expression levels of the indicated mRNAs and proteins in untreated and Ang II-stimulated NRK-52E cells. The cells were rendered quiescent by incubation for 24 h and then infected with the indicated doses of Ad-clusterin-GFP (Ad-Clusterin) or Ad-GFP for 2 h. After incubation for a further 20 h, the cells were incubated with 200 nM Ang II for 4 h. (**A** and **B**) Representative real-time RT-PCR (**A**) and immunoblot (**B**) analyses of the expression levels of PAI-1, type I collagen, and fibronectin. (**C** and **D**) Representative real-time RT-PCR of AT1R (**C**) and immunoblot (**D**) analyses of the expression of levels of AT1R and p-Smad3. The mRNA expression levels were normalized to those of GAPDH and the data are represented as the mean ± SEM of n = 3 independent measurements (n = 3 separate experiments). The protein expression levels were normalized to those of β-actin and data are expressed as the mean ± SEM of n = 3 independent experiments (n = 5 in each group). ^¶^ *P*<0.01, ^**^P<0.05 and ^##^ *P*<0.001 compared with control (untreated cells); ^††^ *P*<0.01, ^‡‡^ *P*<0.05, and ^§§^ *P*<0.001 compared with Ang II alone.

Whether clusterin inhibits Ang II-stimulated expression of profibrotic genes in cultured renal proximal tubular epithelial cells where expression of clusterin is increased by Ang II treatment (Fig. S1) was evaluated by using real-time RT-PCR and immunoblots. Adenovirus-mediated overexpression of clusterin (Ad-clusterin) in the rat kidney proximal epithelium NRK-52E cell line inhibited Ang II-stimulated expression of the mRNAs encoding PAI-1, type 1 collagen, and fibronectin dose dependently (Fig. 2A). Furthermore, immunoblot analyses indicated similar effects of clusterin at the protein level (Fig. 2B). Ad-clusterin also decreased AT1R mRNA (Fig. 2C) and protein (Fig. 2D) levels in a dose-dependent manner. Likewise, overexpression of clusterin decreased p-Smad3 protein levels dose-dependently, suggesting that the renal fibrogenic process was blocked by clusterin overexpression (Fig. 2D). Protein expression of TGF-β, a main activator of Smad3, was increased by Ang II stimulation, but unaffected by overexpression of clusterin (Fig. S2). Taken together with the results of our previous study [23], this finding suggests that clusterin attenuates renal fibrosis through TGF-β-dependent and -independent Smad signaling.

### Adenovirus-mediated overexpression of clusterin ameliorates Ang II-induced renal fibrosis

**Figure 3:**
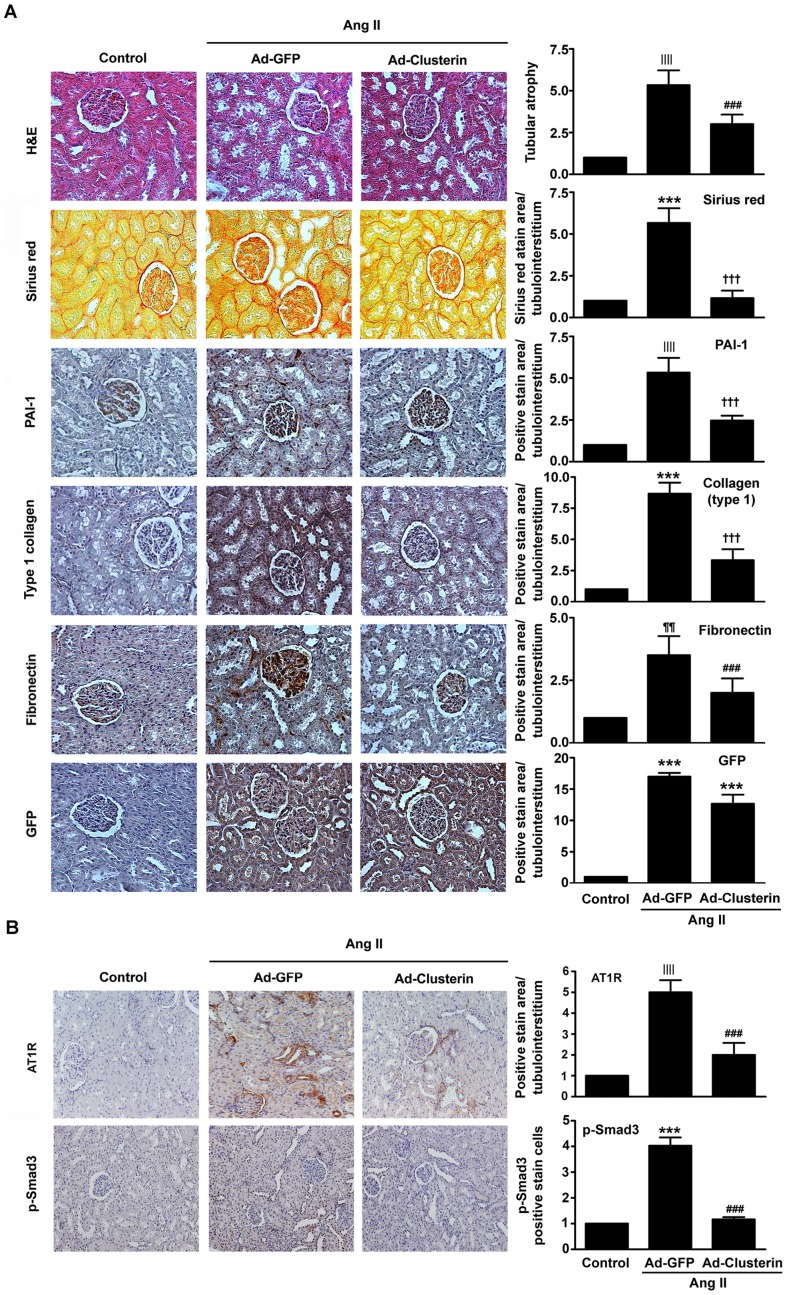
Effects of adenovirus-mediated overexpression of clusterin on Ang II-induced renal fibrosis. Representative images of renal sections from rats infected with Ad-GFP or Ad-Clusterin and treated with Ang II for 14 d. (**A**) The sections were stained with H&E or Sirius red, or were immunostained with antibodies targeting PAI-1, type I collagen, and fibronectin. The number of atrophic tubules was determined by measurement of the abnormal irregular and dilated tubular basement membranes in five random fields of H&E-stained sections under high-power magnification. Areas of positive staining with Sirius red and positive immunostaining with PAI-1, type I collagen, and fibronectin antibodies were quantified by computer-based morphometric analysis. (**B**) The sections were immunostained with an antibody targeting AT1R and p-Smad3. Areas of positive immunostaining were quantified by computer-based morphometric analysis. Data were normalized to the control and are represented as the mean ± SEM of five random fields of each kidney (n = 5 in each group). Original magnification, ×200. ^||||^ *P*<0.01, ^¶¶^ *P*<0.05, and ^***^ *P*<0.001 compared with the control; ^###^ *P*<0.01, and ^†††^ *P*<0.001 compared with Ad-GFP.

**Figure 4:**
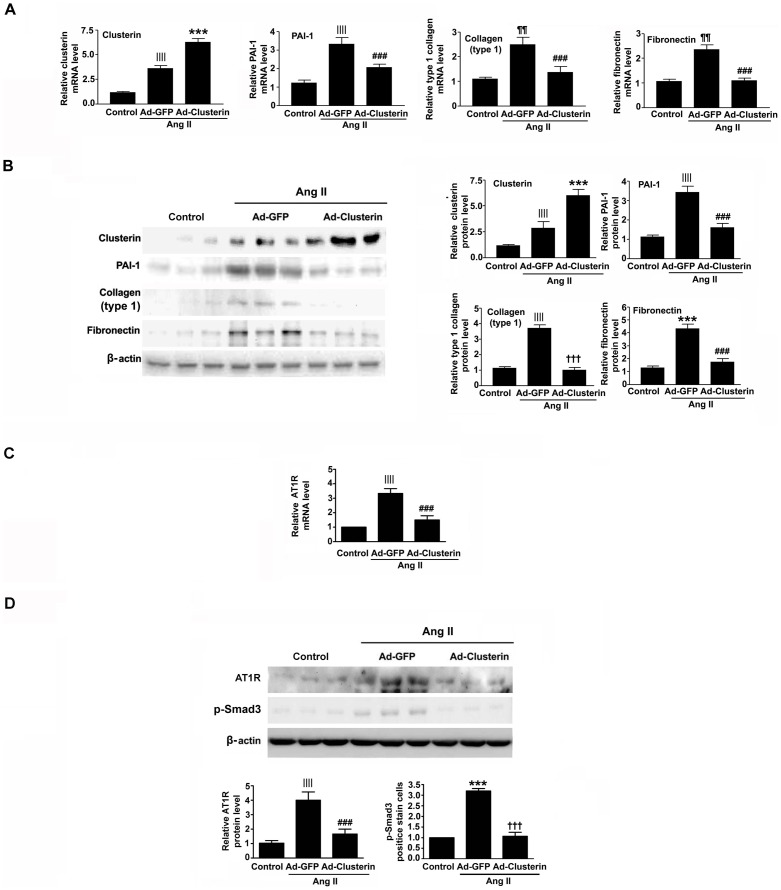
Effects of adenovirus-mediated overexpression of clusterin on Ang II-induced expression of profibrotic genes and AT1R. Rats were infected with Ad-GFP or Ad –clusterin and treated with Ang II for 14 d. (A and B) Representative real-time RT-PCR (A) and immunoblot (B) analyses of the renal expression levels of clusterin, PAI-1, type I collagen, and fibronectin. (C and D) Representative real-time RT-PCR of AT1R (C) and immunoblot (D) analyses of the renal expression levels of AT1R and p-Smad3. The mRNA expression levels were normalized to those of GAPDH and the protein expression levels were normalized to those of β-actin. The data are represented as the mean ± SEM of n = 3 independent measurements (n = 5 in each group). ^||||^ *P*<0.01, ^¶¶^ *P*<0.05, and ^***^ *P*<0.001 compared with the control; ^###^ *P*<0.01 and ^†††^ *P*<0.001 compared with Ad-GFP.

To further evaluate the effect of clusterin against Ang II-induced renal fibrosis *in vivo*, infusion of Ad-clusterin or adenovirus encoding green fluorescent protein (Ad-GFP) into the left kidneys of rats was performed followed by implantation of an osmotic mini-pump containing Ang II. Ad-mediated gene expression was detected by immunohistochemical staining with an anti-GFP antibody (Fig. 3A). Hematoxylin and eosin (H&E) and Sirius red staining of renal sections showed that Ad-clusterin significantly reduced Ang II-induced tubular atrophy and renal fibrosis (Fig. 3A). Immunohistochemical staining also revealed that, after Ang II treatment, mice overexpressing clusterin had significantly lower renal expression levels of PAI-1, type I collagen, and fibronectin than mice infected with Ad-GFP (Fig. 3A). Ang II-stimulated expression of AT1R and p-Smad3 were also significantly decreased by Ad-clusterin (Fig. 3B). The changes in PAI-1, type I collagen, fibronectin, and AT1R mRNA and protein levels were also examined by real-time RT-PCR and immunoblot analyses. Consistent with the immunohistochemical staining results, Ad-clusterin inhibited Ang II-stimulated mRNA and protein expression levels of profibrotic factors (Fig. 4A and B), AT1R and p-Smad3 (Fig. 4C and D).

### Clusterin inhibits Ang II-induced translocation of NF-κB to the nucleus

**Figure 5:**
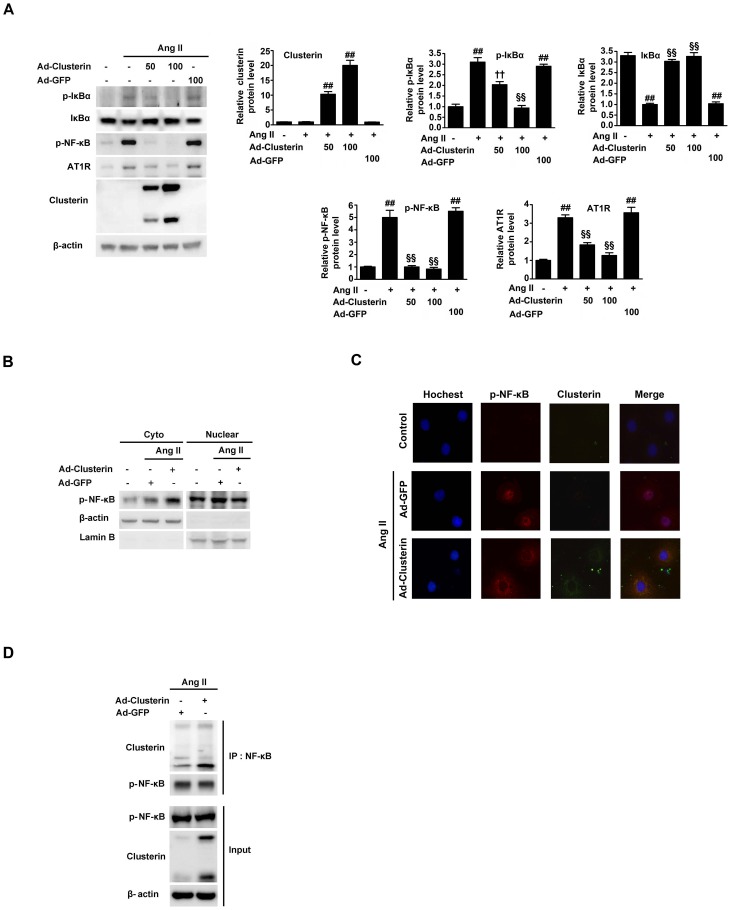
Effect of overexpression of clusterin on Ang II–stimulated NF-κB activation. NRK-52E cells were infected with Ad-clusterin (100 moi) or Ad-GFP (100 moi) as a control after a 24 h serum starvation. After incubation for a further 20 h, the cells were incubated with 200 nM Ang II for 8 h. (A) Representative immunoblot analysis of the expression of AT1R, p-IkBα, IkBα, p-NF-kB and clusterin in Ang II untreated or stimulated NRK-52E cells. β-actin was used as an internal control. ^¶^P<0.01, ^**^P<0.05, and ^##^P<0.001 compared with control (untreated cells); ^††^P<0.01, ^‡‡^P<0.05, and ^§§^P<0.001 compared with Ang II alone. (B) Representative immunoblot of the expression of p-NF-κB in untreated and Ang II-stimulated NRK-52E cells. Nuclear and cytoplasmic (Cyto) proteins were separated by SDS-PAGE and immunoblots were performed using anti-β-actin, anti-Lamin B, and anti-p-NF-κB antibodies. (C) Representative images of immunofluorescent detection of anti-p-NF-κB in untreated and Ang II-stimulated NRK-52E cells. The cellular localizations of p-NF-κB (red), clusterin (green) and nuclei stained with Hoechst (blue) were examined using fluorescence microscopy. Magnification, ×400. (D) Co-immunoprecipitation (IP) of clusterin and NF-κB in Ang II-treated NRK-52E cells. IP was performed using cell lysates and an anti-NF-κB antibody. Samples were analyzed by immunoblotting with anti-clusterin and anti- NF-κB antibodies. As a loading control, β-actin was detected in the input samples.

Because NF-κB up-regulates AT1R [24], [25], we performed immunoblots with an antibody targeting phospho-NF-κB (p-NF-κB) to examine whether clusterin inhibits Ang II-stimulated activation of this transcription factor. NRK-52E cells were incubated for 24 h and then infected with either Ad-clusterin or Ad-GFP for 2 hours. After a further incubation for 20 h, the cells were incubated with 200 nM Ang II for 8 h. Ang II increased the levels of p-NF-κB and p-IκBα, and the increases correlated with decreases in the levels of NF-κB inhibitor, IκBα and AT1R. These increases were abrogated strongly by Ad-clusterin infection (Fig. 5A). Furthermore, overexpression of clusterin increased and decreased the cytosolic and nuclear NF-κB protein levels, respectively (Fig. 5B). The inhibitory effect of clusterin on Ang II-induced nuclear translocation of NF-κB was confirmed by immunostaining and Hoechst staining of NRK-52E cells (Fig. 5C). In addition, immunoprecipitation with an anti-NF-κB antibody and immunoblotting with an anti-clusterin antibody revealed a physical interaction between these two proteins following treatment of cells with Ang II (Fig. 5D).

## Discussion

Our previous study showed that clusterin plays a protective role in the unilateral urethral obstruction-induced renal fibrosis [23]. Here, we extended these findings by evaluation of the role of clusterin in Ang II-induced renal fibrosis, which is more relevant to the pathophysiology of renal diseases. Knockout of clusterin enhanced Ang II-induced expression of profibrotic factors and accelerated Ang II-induced renal fibrosis and damage in mice via upregulation of AT1R. In rats, overexpression of clusterin by intrarenal delivery attenuated Ang II-stimulated expression of PAI-1, matrix proteins, and AT1R. Furthermore, overexpression of clusterin in a renal cell line inhibited Ang II-stimulated NF-κB activation and p-Smad3. These results suggest that clusterin protects against renal fibrosis by downregulating AT1R via blocking nuclear translocation of NF-κB.

The pathogenesis of renal fibrosis is being widely investigated. Beyond its traditional role in the renin-angiotensin system, it has been proposed that Ang II is a main regulator of renal profibrotic factors [5], [8] and an inducer of renal fibrosis that acts by promoting mesangial cell proliferation and hypertrophy, ECM accumulation, and epithelial-mesenchymal transition [26], [27]. These roles of Ang II are mainly mediated via TGF-β [7], [28], which stimulates the expression of profibrotic factors, such as tissue inhibitor of metalloproteinase 1 and PAI-1, and thereby accelerates the accumulation of ECM proteins [4], [29]. In addition, recent studies have demonstrated that infusion of Ang II induces injuries in tubulointerstitium manifested by atrophy and dilatation of tubules [30], which ultimately results in interstitial fibrosis. Conversely, ACE inhibitors [31], [32], AT1R antagonists [33], TGF-β neutralizing antibodies [34] and TGF-β suppression by siRNA [35] attenuate renal fibrosis. Here, Ang II-induced renal fibrosis was exacerbated or prevented by the loss or overexpression of clusterin, respectively. In addition, the increase in the level of TGF-β induced by Ang II treatment was not affected by overexpression of clusterin, although the p-Smad3 level was decreased. Besides TGF-β, many mediators, such as advanced glycation end-products and Ang II, can activate Smad3, which acts as a signal integrator in the pathophysiological process leading to kidney disease [36]–[38]. In our previous study, intrarenal infusion of clusterin reduced the level of renal fibrosis and decreased PAI-1, matrix protein expression, and TGF-β/Smad3 activity in UUO mice [23], indicating that clusterin plays a protective role in renal fibrosis. Collectively, these findings suggest that Ang II is central to the development of renal fibrosis and that clusterin is an important regulator of this process.

The majority of the unfavorable events of Ang II in the kidney are mainly mediated via AT1R rather than AT2R [39] and several lines of evidence indicate that AT1R is indispensable to Ang II-induced renal fibrosis. Blocking AT1R diminishes renal tubular damage and decreases PAI-1 [40], [41] and fibronectin expression [42]. A number of *in vivo* and *in vitro* studies, such as those using rat mesangial [13], [43] and mononuclear cells [44] have shown that NF-κB is activated by Ang II. Followed by translocation into the nucleus, activated NF-κB upregulates transcription of its target genes, including AT1R [24], [25]. In the present study, we found that clusterin decreased the level of p-NF-κB. This was related to the inhibition of nuclear translocation of p-NF-κB resulting from the interaction between clusterin and p-NF-κB, which led to decreased AT1R expression and fibrosis.

In summary, clusterin attenuates Ang II-induced renal fibrosis by downregulating AT1R; this action is mainly mediated by inhibition of NF-κB nuclear translocation. This study suggests that clusterin could be targeted for the prevention and treatment of renal fibrosis.

## Supporting Information

Figure S1 **The increase of clusterin expression in the renal tubular area after Ang II treatment.** Immunohistochemical stain for clusterin in kidneys from mice with or without Ang II treatment. Data from the Ang II-treated kidneys were normalized to the control ( = 1, wild-type mice) and in the bar graph were expressed as fold increases in clusterin expression relative to the control. Data are the mean ± SEM of five random fields of each kidney (n = 5 in each group). ^†^P<0.001 compared with control. Original magnification, ×400.
(TIF)

Figure S2 **The effect of clusterin on Ang II-stimulated TGF-β expression.** Representative Western blots of the expression of TGF-β in Ang II–stimulated NRK-52E cells. Cells were infected with Ad-clusterin-GFP (Ad-Clusterin) or Ad-GFP for 2 h. After incubation for a further 20 h, the cells were incubated with Ang II (200 nM) for 4 h. ^¶^ *P*<0.01, ^##^ *P*<0.001 compared with control (untreated cells).
(TIF)
