# Supplementary figures and images for: Clusterin/Apolipoprotein J Attenuates Angiotensin II-Induced Renal Fibrosis
Source: PLoS One. 2014 Aug 22;9(8):e105635. doi: 10.1371/journal.pone.0105635 (PMC4141810; doi:10.1371/journal.pone.0105635)

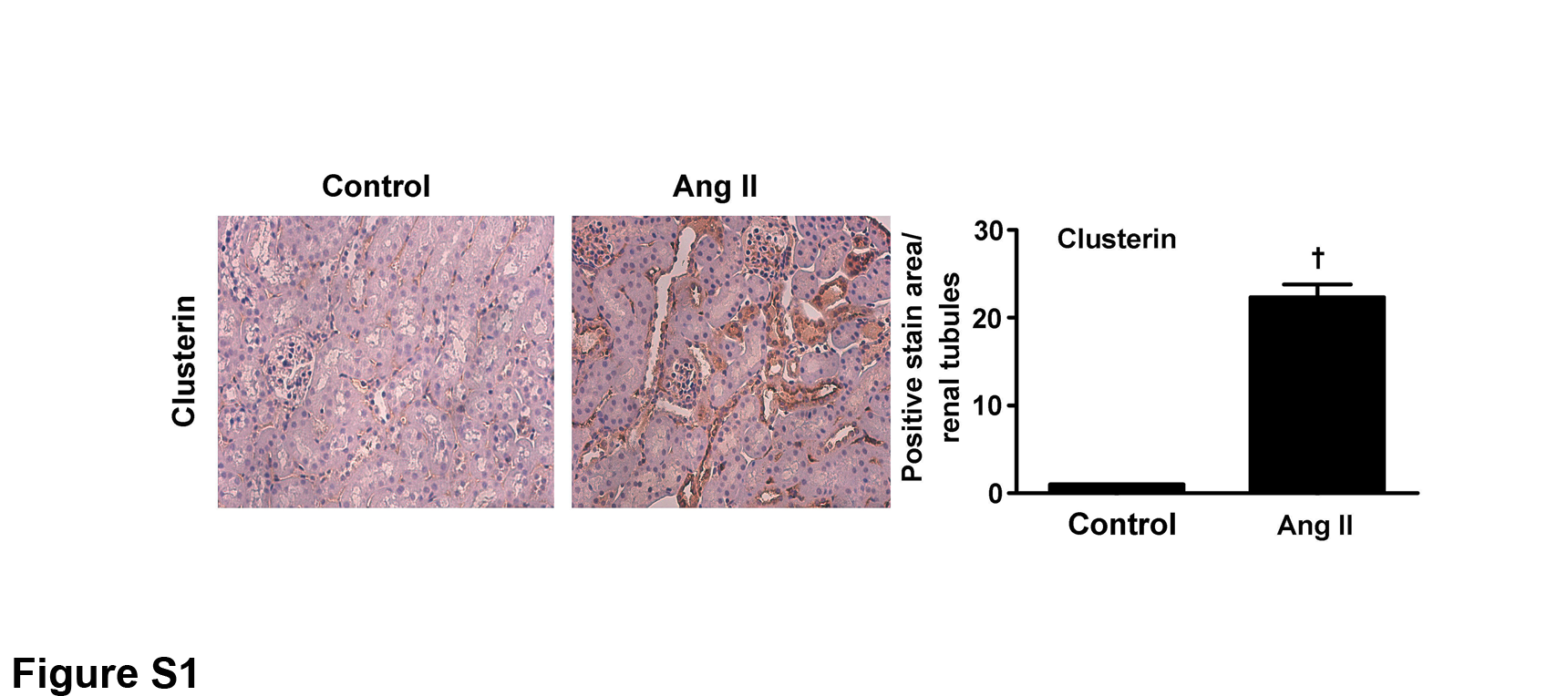

Supplement: Figure S1 — The increase of clusterin expression in the renal tubular area after Ang II treatment. Immunohistochemical stain for clusterin in kidneys from mice with or without Ang II treatment. Data from the Ang II-treated kidneys were normalized to the control ( = 1, wild-type mice) and in the bar graph were expressed as fold increases in clusterin expression relative to the control. Data are the mean ± SEM of five random fields of each kidney (n = 5 in each group). †P<0.001 compared with control. Original magnification, ×400. (TIF) [file pone.0105635.s001.tif]

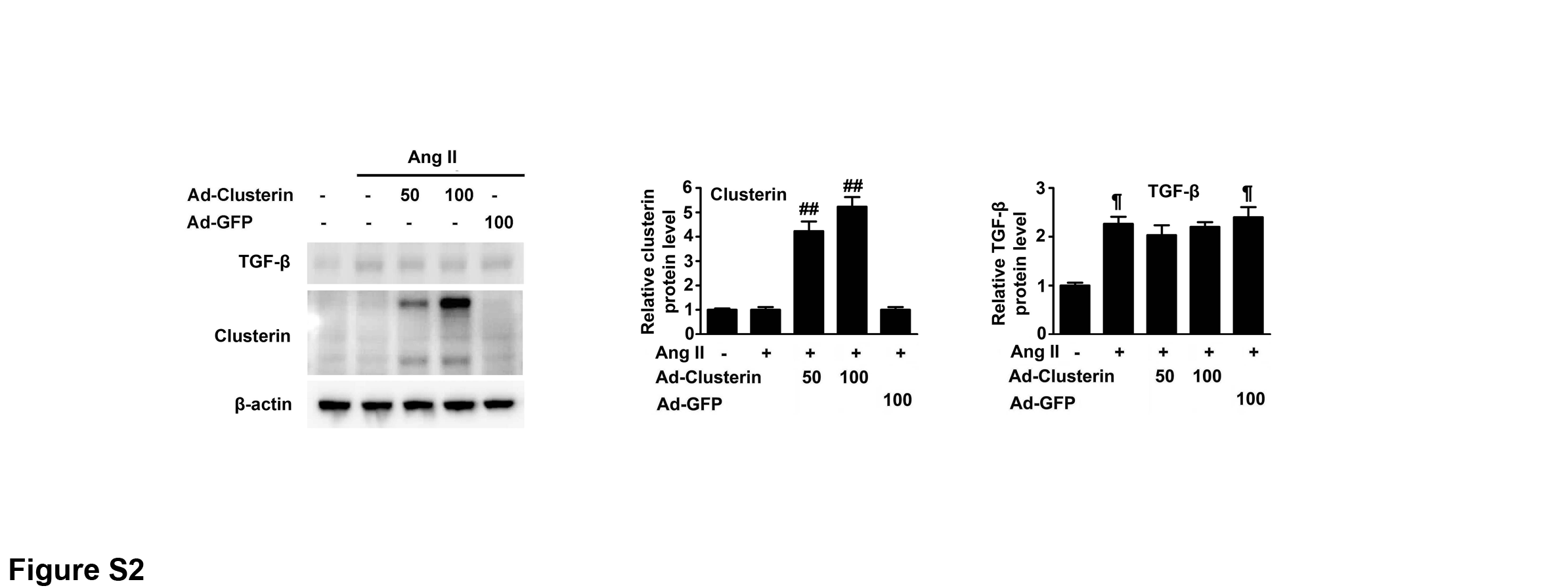

Supplement: Figure S2 — The effect of clusterin on Ang II-stimulated TGF-β expression. Representative Western blots of the expression of TGF-β in Ang II–stimulated NRK-52E cells. Cells were infected with Ad-clusterin-GFP (Ad-Clusterin) or Ad-GFP for 2 h. After incubation for a further 20 h, the cells were incubated with Ang II (200 nM) for 4 h. ¶ P<0.01, ## P<0.001 compared with control (untreated cells). (TIF) [file pone.0105635.s002.tif]
